# Ultraviolet Radiation-Induced Cytogenetic Damage in White, Hispanic and Black Skin Melanocytes: A Risk for Cutaneous Melanoma

**DOI:** 10.3390/cancers7030852

**Published:** 2015-08-14

**Authors:** Amrita Dasgupta, Meena Katdare

**Affiliations:** 1 Hampton University Skin of Color Research Institute, Hampton, VA 23668, USA; E-Mail: ritu.adg@gmail.com; 2 Department of Dermatology, Eastern Virginia Medical School, Norfolk, VA 23507, USA

**Keywords:** cutaneous melanoma, Hispanics, melanocytes, ultraviolet radiation, DNA damage, cytogenetic damage, melanin pigmentation, skin, ethnicity

## Abstract

Cutaneous Melanoma (CM) is a leading cause of cancer deaths, with reports indicating a rising trend in the incidence rate of melanoma among Hispanics in certain U.S. states. The level of melanin pigmentation in the skin is suggested to render photoprotection from the DNA-damaging effects of Ultraviolet Radiation (UVR). UVR-induced DNA damage leads to cytogenetic defects visualized as the formation of micronuclei, multinuclei and polymorphic nuclei in cells, and a hallmark of cancer risk. The causative relationship between Sun exposure and CM is controversial, especially in Hispanics and needs further evaluation. This study was initiated with melanocytes from White, Hispanic and Black neonatal foreskins which were exposed to UVR to assess their susceptibility to UVR-induced modulation of cellular growth, cytogenetic damage, intracellular and released melanin. Our results show that White and Hispanic skin melanocytes with similar levels of constitutive melanin are susceptible to UVR-induced cytogenetic damage, whereas Black skin melanocytes are not. Our data suggest that the risk of developing UVR-induced CM in a skin type is correlated with the level of cutaneous pigmentation and its ethnic background. This study provides a benchmark for further investigation on the damaging effects of UVR as risk for CM in Hispanics.

## 1. Introduction

Cutaneous Melanoma (CM), the 8th most common cancer in the United States [1] and a leading cause of cancer deaths among young adults, is a type of skin cancer that originates from the malignant transformation of cells called melanocytes in the skin [2,3]. Although the age-adjusted incidence rates (per 100,000) for melanoma are lower among Hispanics and Blacks (4.5 and 1.0, respectively) compared with non-Hispanic Whites (21.6) [4], reports indicate that melanoma incidence among Hispanics in certain regions of the U.S. has risen [5,6,7] with limited information of its risk factors.

The human skin exhibits a vast variation in the color of skin which is a consequence of the quantity and the quality of melanin pigment in melanocytes [8]. Melanin is synthesized, packaged and distributed in specific ovoid organelles called melanosomes [8,9,10]. Melanin is produced by the oxidation of the amino acid tyrosine, followed by polymerization [8]. There are three basic types of melanin: eumelanin (darker, brown/black), pheomelanin (lighter, red/yellow), and neuromelanin (dark) [8,11]. The relative amount of eumelanin and pheomelanin is a key determinant of color-based ethnic diversification in humans [12]. Darkly pigmented skin typically contains larger and greater number of melanosomes with higher levels of melanin (eumelanin). Lightly pigmented skin is associated with smaller and less dense melanosomes [11]. Ultraviolet radiation (UVR) from the sunlight has been considered to be one of the primary carcinogenic risk factors responsible for the initiation of CM [2,13,14,15,16,17,18]. Melanin is suggested to render photoprotection from UVR and hence constitutive levels of melanin in skin may influence UVR-induced CM [19,20]. In addition to differences in constitutive skin pigmentation (relative proportions of eumelanin vs pheomelanin), other risk factors that can influence UVR-induced CM include inefficiency in DNA repair mechanisms [21], genetic variants in pigmentation genes (*MC1R*, *ASIP*, *TYR* and *TYRP1*) [22,23,24] and mutations in genes like *BRAF* [25], *NRAS* [26], *KIT* [27] and mutations in c-Met [28,29].

The UVR that penetrates the atmosphere is composed of, long wavelength ultraviolet A (UVA) (320–400 nm) and short wavelength ultraviolet B (UVB) (280–320 nm). Both UVA and UVB exposures can promote the formation of oxidized DNA bases such as 8-oxo-7,8-dihydro-2′-deoxyguanosine(8-oxodG) [30]. UVA exposure results in the generation of reactive oxygen species (ROS) which induce oxidative damage to DNA [31,32,33] and formation of photoproducts such as cyclobutane pyrimidine dimers (CPD) [34,35]. UVB exposure induces DNA double-strand breaks [36] due to formation of DNA photoproducts (CPDs and pyrimidine (6-4) pyrimidine) [12,37,38]. The primary function of cutaneous melanin is to render photoprotection to UVR [34,39] since melanin absorbs sunlight and scavenges free radicals generated by UVR [40]. However, reports have suggested that melanin (particularly pheomelanin) and its intermediates augment UVA-induced generation of ROS and oxidative DNA damage [41,42]. The reported antagonistic roles of melanin on exposure of skin to either UVA or UVB suggest that the relationship between skin pigmentation and UVR-induced photodamage is complex and needs critical evaluation. Additionally, it has been shown [43,44] that melanocytes have a lower DNA-repair capacity intrinsically, making them more susceptible to UVR-induced cytogenetic defects.

UVR-induced carcinogenesis manifests itself with cytogenetic damages resulting in alterations in nuclear structure leading to increases in nuclear size, deformities in nuclear shape, and changes in the internal organization of the nucleus [45,46]. The alterations reported include the observation of micronuclei (MN) [47,48,49], multinucleated (MLTN) [37] and/or polymorphic nuclear cells (PMN) cells [50]. MN represent fragmented genetic material that fails to be incorporated into the daughter nuclei during cell division and appear as small additional nucleus [51]. MN formation occur due to DNA double-strand breaks that are a consequence of UVR-induced pyrimidine dimers [36]. These cytogenetic defects observed as MN, MLTN and PMN are known to serve as biomarkers of cancer risk in human [37,45].

Ethnicity is grossly categorized based on the color of the skin (White, Hispanic and Black). Skin of color varies between different ethnic groups as well as between individuals within the same group. This variation therefore can lead to differences in the degree of susceptibility to UVR-induced DNA damage within an ethnic group. One such large ethnic group with a vast variation in skin pigmentation is the Hispanic population in the U.S. [52]. As stated earlier CM incidence among Hispanics has risen significantly [5,6] with limited knowledge of its etiology. In the state of California, where Hispanics comprise almost 40% of the population (~12 million), increase in CM with worst prognosis, metastasis and high mortality have been reported [53,54,55]. An overall increase in the melanoma incidence rates (1% in Hispanic men and 3.4% in Hispanic women) among Hispanics has also been reported in the state of Florida [6]. Though no significant increase in the incidence of CM in the state of Connecticut was reported, a significantly higher proportion of advanced stage melanoma was reported in Hispanics [56]. Although the increase in incidence of melanoma in Hispanics has been theoretically shown to be positively correlated with overexposure to UVR [52], the effects of UV exposure in initiation of CM is poorly understood [57] and remains unexplored. Hence the reports of the alarming rise in the incidence rates of CM in Hispanics warrant an urgent unmet need to investigate UVR as a carcinogenic risk factor [58]. This study was designed to investigate the differential response and susceptibility of normal neonatal foreskin melanocytes (White, Hispanic and Black) as a model to elucidate UVR-induced cytogenetic damage as a risk factor to CM.

## 2. Results

### 2.1. Melanin in Melanocytes from Three Ethnic Individuals

**Figure 1:**
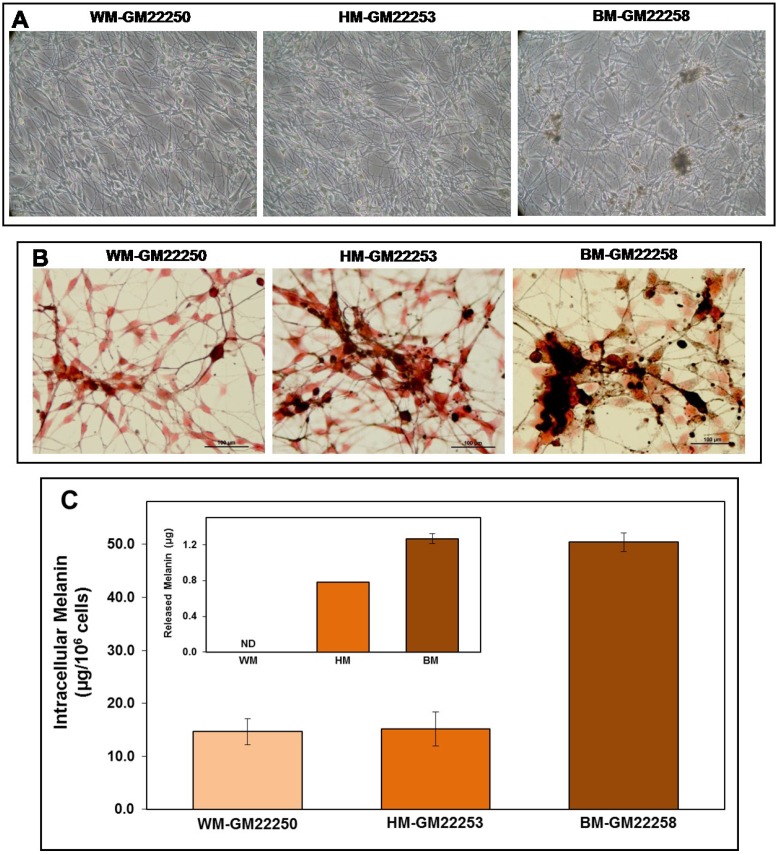
(**A**) Phase contrast (20×) images of cultured melanocytes; White (WM-GM22250), Hispanic (HM-GM22253) and Black (BM-GM22258); (**B**) Fontana-Masson staining (40×) for melanin; White (WM-GM22250), Hispanic (HM-GM22253) and Black (BM-GM22258) melanocytes in culture; (**C**) Spectrophotometric quantitation of the constitutive levels of intracellular and released melanin (inset) in White (WM-GM22250), Hispanic (HM-GM22253) and Black (BM-GM22258) melanocyte cultures at log phase of growth. Error bars show the standard deviation obtained from *n* = 3. ND: non-detectable.

**Figure 2:**
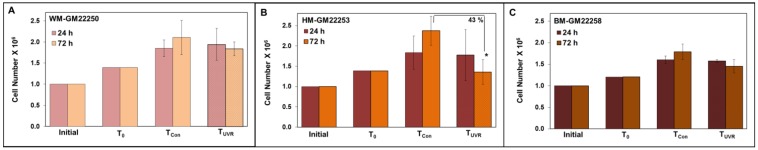
Susceptibility of UVR to growth of normal skin melanocytes; (**A**) White, (**B**) Hispanic and (**C**) Black. Graphs indicate cell number at the time of plating (Initial); Time of UV exposure (T_0_); Control (T_Con_); post UV exposure (T_UVR_). Error bars show the standard deviation obtained from *n* = 3. (* *p* < 0.05).

To estimate the levels of intracellular melanin in the three ethnic categories of melanocytes, all three types of melanocytes were maintained under identical culture conditions. Figure 1A shows the three types of melanocytes in culture, exhibiting different amounts of intracellular melanin. Presence of melanin was confirmed by Fontana-Masson staining as shown in Figure 2B. Images show that Black skin melanocytes (BM-GM22258) have a significantly higher constitutive level of intracellular as well as released melanin (melanosomes) compared to that in White (WM-GM22250) and Hispanic (HM-GM22253) skin melanocytes. Spent media of cultures and attached melanocytes were harvested at log phase of growth to estimate the constitutive levels of released (Figure 1C inset) and intracellular melanin respectively (Figure 1C). Our results show that White and Hispanic skin melanocytes used in this study have almost the same level of intracellular melanin, implying that HM-GM22253 melanocyte cell line was derived from a lightly pigmented Hispanic subject. Interestingly, Hispanic and Black skin melanocytes in culture showed significant release of melanin (Figure 1C inset) as evaluated from spent media. No significant release of melanin was detectable in White skin melanocytes.

### 2.2. Effect of UVR on Growth of Melanocytes in Culture

All three types of normal melanocytes did not show any significant difference in their growth rates under our standard culture conditions. Normal melanocytes from White, Hispanic and Black skin, with different levels of melanin were exposed to UVR that covered the UV spectrum similar to solar radiation (280–400 nm) rather than only UVA or UVB. The response of UVR on growth of melanocytes was determined at 24 h and 72 h post UV exposure. Our results (Figure 2A–C) clearly indicate that the single dose of UVR used in this study was not cytotoxic as the cell number did not alter at 24 h post UV exposure for either type of melanocyte cell line. While there was no change in the cell number of White (Figure 2A) and Black (Figure 2C) skin melanocytes at 72 h post UV exposure, Hispanic (Figure 2B) skin melanocytes showed a significant (43%) reduction in overall cell number. Although, it is important to note that the total number of Hispanic skin melanocytes at 72 h post UVR was not less than the melanocyte number at the time when cultures were exposed to UVR (T_0_). 

**Figure 3:**
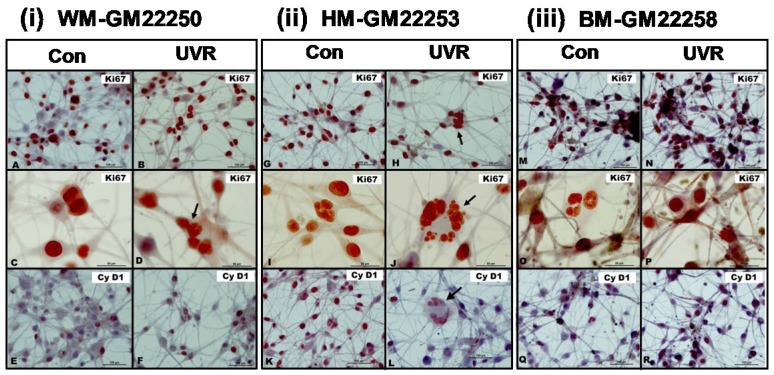
Effect of UVR on the expression of proliferation markers Ki67 and Cyclin D1 (Cy D1) on normal skin melanocytes; (**i**) White, (**ii**) Hispanic and (**iii**) Black at 72 h post UV exposure. Images shown in **A**–**B**, **E**–**F**, **G**–**H**, **K**–**L**, **M**–**N**, **Q**–**R** were obtained at 40× and **C**–**D**, **I**–**J**, **O**–**P** at 100× oil immersion. Black arrows indicate the detection of MN, MLTN and/or PMN only in UV exposed White and Hispanic skin melanocytes.

Cellular growth (assessed by the cell number) of UV exposed melanocytes was further correlated with expression of molecular markers of cell proliferation, Ki67 and Cyclin D1 (Cy D1). The expression of Ki67 has been suggested to be an excellent marker for determining the fraction of proliferating cells in a given population [59]. Cy D1 has been implicated to control cell cycle progression and is expressed when the cells have the signal to proliferate [60]. Although Ki67 and Cy D1 are expressed at different stages of cell cycle progression, their overexpression has been implicated in the oncogenic transformation of cells. Our results demonstrated a reduction in expression of proliferation markers Ki67 and Cy D1 in Hispanic skin melanocytes (Figure 3ii (G–H, K–L)) that correlates with the decrease in cell number observed at 72 h post UVR. No significant reduction in the expression of Ki67 and Cy D1 was observed in the melanocytes from White (Figure 3i (A–B, E–F)) or Black (Figure 3iii (M–N, Q–R)) skin which correlates with the insignificant reduction in cell number observed at 72 h post UVR. To evaluate UVR-induced apoptotic cell death of melanocytes, expression of p53 was used as a molecular marker. All three types of melanocytes did not show any cell death (morphological criteria and p53 expression) at 24 h and 72 h post UVR (Supplementary Figure S1) reiterating the fact that the dose of UVR used in this study was not cytotoxic to melanocytes.

### 2.3. Susceptibility of Melanocytes to UVR-Induced Cytogenetic Damage

**Figure 4:**
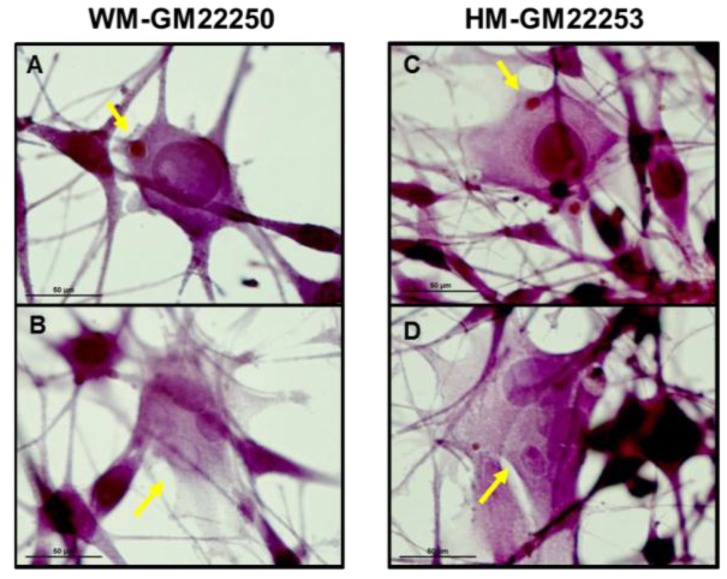

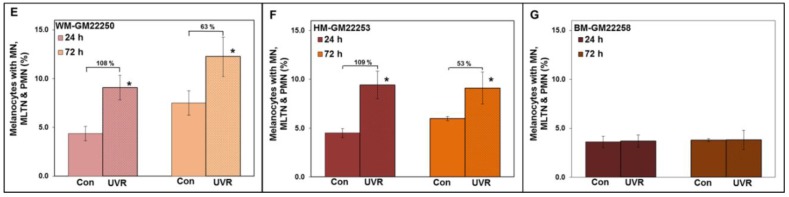
UVR-induced formation of melanocytes containing MN, MLTN and PMN. Representative images (100× oil immersion) of HE stained, UV exposed, White skin melanocytes containing MN (**A**) and MLTN/PMN (**B**) as well as Hispanic skin melanocytes containing MN (**C**) and MLTN/PMN (**D**), at 72 h post UVR (indicated by yellow arrows). Quantitation of White (**E**), Hispanic (**F**), and Black (**G**) skin melanocytes containing MN, MLTN and PMN at 24 h and 72 h post UVR. Error bars show the standard deviation obtained from *n* = 4. (* *p* < 0.05).

The susceptibility of three types of melanocytes to UVR was evaluated by quantifying the number of melanocytes with MN, MLTN and PMN. The formation of MN, MLTN and PMN cytogenetic defects serve as surrogate markers of cancer risk. Our results (Figure 4) illustrate that both White (Figure 4E) and Hispanic (Figure 4F) skin melanocytes showed a significant increase in the number of melanocytes containing MN, MLTN and PMN at 24 h and 72 h post UV exposure indicating UVR-induced DNA damage leading to cytogenetic defects (also see Figure 3D,J). The extent of cytogenetic damage observed at 24 h post UV exposure persisted to be significantly high even at 72 h post UVR. Black (Figure 4G) skin melanocytes did not show any significant change in the number of melanocytes containing MN, MLTN or PMN indicating that darkly pigmented melanocytes are photoprotected from UVR at the dose used in this study.

### 2.4. Modulation of Melanin Content Post UVR

**Figure 5:**
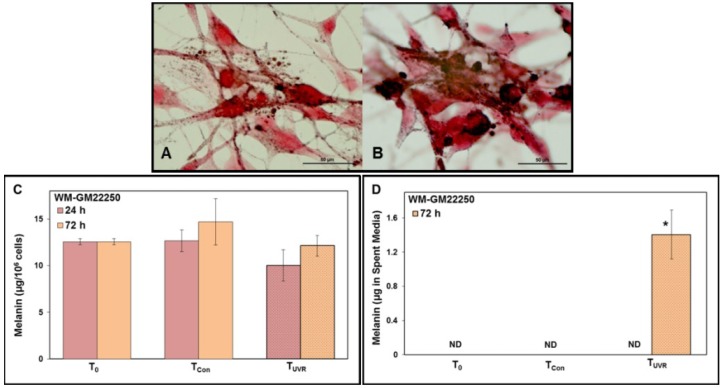

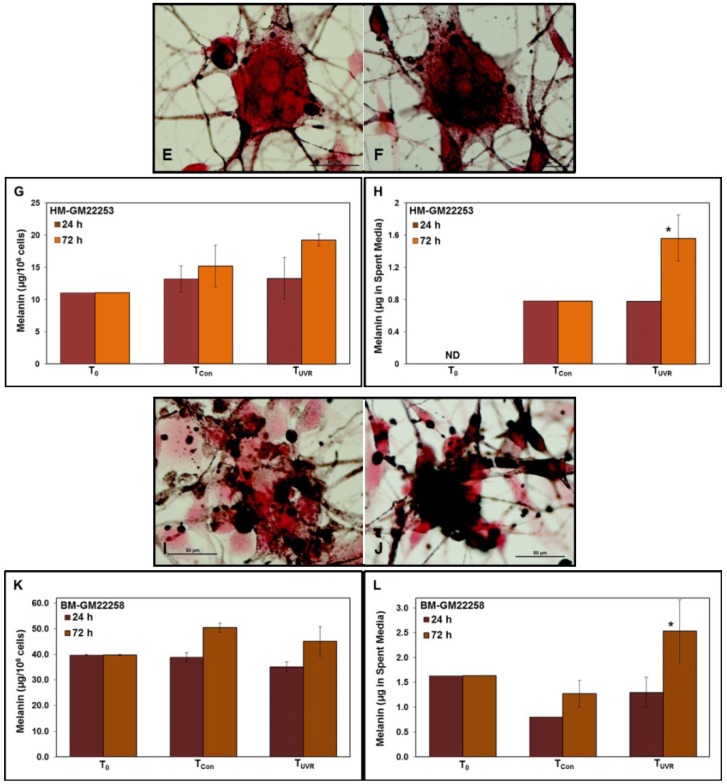
Images (100× oil immersion) of Fontana-Masson staining of melanocytes from White (**A**, Con; **B**, UVR), Hispanic (**E**, Con; **F**, UVR) and Black (**I**, Con; **J**, UVR) skin at 72 h post UVR for visualization of intracellular and released melanin. Spectrophotometric quantitation of intracellular and released melanin for White (**C** and **D** respectively), Hispanic (**G** and **H** respectively) and Black (**K** and **L** respectively) skin melanocytes at 24 h and 72 h post UV exposure. Graphs indicate melanin content at UV exposure (T_0_), Control (T_Con_) and post UV exposure (T_UVR_). Error bars show the standard deviation obtained from *n* = 3. ND: non-detectable. (* *p* < 0.05).

To investigate the effect of UVR on melanin content, the intracellular and released melanin was estimated. Our results show that UV exposure to White (Figure 5C), Hispanic (Figure 5G) and Black (Figure 5K) skin melanocytes did not alter the level of intracellular melanin compared to their corresponding controls (levels remained unaltered to that at the time of UV exposure; T_0_) at 24 h post UV exposure. At 72 h post UVR, Hispanic (Figure 5G) skin melanocytes exhibited a UV triggered increase in the level of intracellular melanin (non-significant) compared to the control (level higher than that at time of exposure; T_0_). Exposure to UVR to all three types of skin melanocytes triggered a significant increased level of released melanin as detected in the spent media at 72 h post UVR. This increase was higher in Black (Figure 5L) skin melanocytes compared to White (Figure 5D) or Hispanic (Figure 5H) skin melanocytes. The data from the quantitative estimation of intracellular and released melanin were corroborated with visual confirmation using Fontana-Masson staining (Figure 5A,B,E,F,I,J).

## 3. Discussion

In the present study, the susceptibility of normal neonatal foreskin melanocytes from three different ethnic individuals to UVR was assessed. Several key observations were made: (1) Constitutive levels of intracellular and released melanin was significantly higher in Black skin melanocytes compared to White and Hispanic skin melanocytes evaluated at the same level of their growth phase; (2) White and Hispanic skin melanocytes used in this study have the same levels of intracellular melanin, suggesting that HM-GM22253 is derived from a lightly pigmented Hispanic subject; (3) susceptibility to UVR-induced modulation of growth (72 h post UVR) was only observed in Hispanic skin melanocytes as a decrease in the cell number correlating with reduction in expression of proliferation markers Ki67 and Cy D1; (4) White as well as Hispanic skin melanocytes showed a significant increase in the number of melanocytes containing MN, MLTN and PMN at 24 h and 72 h post UV exposure; (5) Interestingly UV exposed Hispanic skin melanocytes showed an increased level of intracellular melanin as well as released melanin. UVR did not induce any change in the level of intracellular melanin in White and Black skin melanocytes; however, it did lead to a significant amount of released melanin in cultures of both cell lines.

There are very limited reports on the effect of the entire range of UVR from the Sun on either human skin [39] or melanocytes in culture [61] and, in particular on skin from different ethnic origins [39]. While the molecular responses of exposure to either UVA or UVB individually on melanocytes has been studied extensively [24,37,61,62,63,64,65,66], simultaneous exposure to all the spectral components of solar UVR may trigger a broader range of DNA lesions and oxidative damage [67]. Therefore the UVR source used in this study, covering the spectrum of solar UVR, might have led to a differential modulation of cytogenetic damage and molecular changes in the three types of skin melanocytes than using a source that emitted only specific wavebands of UVR. Furthermore, most studies cited above have used normal melanocytes from either lightly or darkly pigmented individuals or both to evaluate their response to either UVA [37,65,66] or UVB [62,63] exposure but not from cultured melanocytes that are derived from skin of different ethnic background as used in this study.

The present study was initiated assuming that an increasing order of constitutive melanin content in melanocytes from White, Hispanic and Black skin will influence the susceptibility to UVR. Interestingly, estimation of constitutive levels of intracellular melanin content of the three types of melanocytes showed that the Hispanic skin melanocytes were derived from a lightly pigmented individual. The skin pigmentation is influenced by the synthesis of melanin and its packaging in melanosomes and the transfer of melanosomes to keratinocytes [68,69]. The inherent heterogeneity of melanin content within melanosomes and the melanosomal distribution in melanocytes in skin of different ethnic origin is a consequence of their genetic background [70]. When considering the final color of the skin, the relative proportions of different types of melanin (insoluble eumelanin and alkali-soluble pheomelanin) are also important [8,11,12]. In this study, the Fontana-Masson based visualization of melanin distribution showed that, constitutively, Black skin melanocytes contained numerous single large melanosomes which were intensely melanotic, while both White and Hispanic skin melanocytes showed smaller and less dense melanosomes. Within individual melanocytes melanin accumulates as nuclear “caps” that is suggested to protect from UVR-induced DNA damage [11,12]. These characteristic features have been well-documented in previous reports in lightly and darkly pigmented melanocytes [11,69]. Visualization of Hispanic skin melanocytes showed a marginally higher intensity of melanin compared to White skin melanocytes, although the quantitative estimation of intracellular melanin did not show any difference. This could be due to the presence of more eumelanin compared to pheomelanin in Hispanic skin melanocytes and needs further evaluation. In Black skin melanocytes both types of melanin are probably present in significant amounts. Since it is known that the different types of melanin have differential solubility [12], in the current study the method of alkaline/heat extraction might have resulted in the incomplete extraction of total melanin from the melanocytes. Nevertheless, our results highlight that it is not correct to assume that all individuals from a particular ethnicity will have uniform melanin based color of skin and that the entire ethnic group will have uniform susceptibility to UVR-induced photodamage.

Exposure to a single dose of UVR (200 mJ/cm^2^) did not result in any significant cytotoxicity in all the three types of melanocytes as evaluated by the cell number at 24 h post UVR. As mentioned earlier, the UVR was given when the cells were already in the log phase of growth indicating that they were actively dividing. Our results showed a slight reduction in the expression of Ki67 and Cy D1 in all three types of melanocytes at 24 h post UV exposure (data not shown). This suggests alteration in cell cycle (like cell cycle arrest) which is probably a response to the DNA damage caused by UVR [37,63]. Such a cell cycle delay can function as a checkpoint to protect the cell’s genetic integrity. Since there was no change in cell number at this time point, it was imperative to check if this effect persisted when the UV treated cells were allowed to grow and divide further. The cell number at 72 h post UVR in the White and Black skin melanocytes showed an insignificant decrease, whereas Hispanic skin melanocytes showed significant response (cell number was same as T_0_, but less than T_Con_) to UVR-induced modulation of growth. Overall, at 72 h post UVR, there was a reduction in expression of Ki67 and Cy D1 in all three types of melanocytes but this reduction was significant in Hispanic skin melanocytes indicating that in spite of similar constitutive levels of melanin, susceptibility to UVR of White and Hispanic skin melanocytes is probably dependent on the genetic background and not just photoprotection by melanin.

DNA absorbs both UVA and UVB that leads to significant alterations in its function and structure [67]. A previous study on the effect of UVR on skin from different ethnic origin suggested that in addition to the immediate UVR-induced DNA damage and mutated genes, the efficiency of DNA repair is equally important in the induction of photocarcinogenesis [39]. The extent of UVR-induced DNA damage (formation of MN, MLTN and PMN) observed in Hispanic skin melanocytes was similar to that observed in the White skin melanocytes at 24 h as well as 72 h post UV exposure. The similarity in susceptibility to UVR-induced cytogenetic damage exhibited by both White and Hispanic skin melanocytes used in this study could be explained by the similarity in the constitutive levels of pigmentation. It is important to note that the UVR-induced DNA damage was significantly lower in both White and Hispanic skin melanocytes at 72 h post UVR compared to that at 24 h post UVR (Figure 4) suggesting induction of repair mechanism. Black skin melanocytes did not show any significant increase in MN, MLTN or PMN formation post UVR. There are two plausible explanations for this observation; Black skin melanocytes are photoprotected due to the elevated level of constitutive melanin and hence have very minimal DNA damage and/or they have an efficient DNA repair mechanism that minimizes the passage of deleterious mutations to daughter cells [39,71]. In our study, the former explanation is more likely since DNA repair induced reduction in cell division was not observed as exhibited by an insignificant change in Black melanocyte cell number post UVR.

Earlier reports have shown that UV exposure and subsequent DNA damage can stimulate melanogenesis in cultured melanocytes [72,73,74]. To evaluate the effect of UVR on melanin content in three different types of melanocytes, the intracellular melanin as well as released melanin was quantified. All the three types of melanocytes did not show any significant change in their intracellular melanin content at 24 h post UVR compared to the intracellular melanin in melanocytes at the time of UV exposure (T_0_, Figure 5). Unaltered levels of melanin at 24 h post UVR could be a consequence of melanocytes unaffected by UV exposure and continued in their log phase of growth. An increase in the number of differentiated melanocytes would have resulted in increased levels of melanin. No significant difference in intracellular melanin was observed in melanocytes of White and Black skin even at 72 h post UVR. Interestingly, Hispanic skin melanocytes did show slight increase in their intracellular melanin content at 72 h post UVR (T_UVR_, Figure 5G) with overall cell number remaining similar to that at the time of UV exposure (T_0_, Figure 2B), though the overall cell number was less than the corresponding untreated controls (T_Con,_ Figure 2B). This reflects that UV exposure altered the Hispanic melanocyte population towards higher proportion of differentiated melanocytes compared to proliferating melanocytes thereby leading to increased level of intracellular melanin. A previous report on skin from moderately pigmented Hispanic individual has shown a slight increase in melanin content post UV exposure [39]. To the best of our knowledge there is no report with focus on the effect of UVR either on Hispanic melanocytes or skin which is lightly pigmented.

It is well known that melanocytes transfer melanin in melanosomes through their dendritic contacts to keratinocytes which offers photoprotection [12,74,75]. In our study the released melanin quantified from spent media of the cultured melanocytes was isolated before and after UVR to be used as the indicator of melanin released into the media. Interestingly, all three types of melanocytes showed a significant increase in the levels of released melanin at 72 h post UV exposure (Figure 5D,H,L). These results were further corroborated with visual evidence from the Fontana-Masson stain used in this study (Figure 5). Our results showed an increased level of melanin released into the spent media post UVR, which represents the *in situ* phenomenon of transfer of melanin in melanosomes by melanocytes to keratinocytes in response to UVR [11,74]. The amount of released melanin was much higher in Black skin melanocytes in response to UV exposure suggesting that higher melanin pigmentation (intracellular) as well as facultative pigmentation (released melanin) offer better protection for UVR-induced DNA damage.

In spite of the similarity in their pigmentation and UVR-induced susceptibility to DNA damage of White and Hispanic skin melanocytes, the differential response of Hispanic skin melanocytes to UVR-induced growth and expression of Ki67 and Cy D1 at 72 h post UV exposure (see earlier) and the modulation of melanin content is a reflection of the variability in the genetic background.

Previous reports have shown that UV exposure results in activation and altered expression of tumor suppressor protein p53 [76,77], influencing proliferation and apoptosis [78,79]. A p53-dependent cell cycle arrest observed as its translocation to nucleus is shown to play a significant role in repair of UVR-induced DNA damage [78,80,81]. However, we did not observe any UVR-induced modulation of p53 either in its expression or its translocation to nucleus in the three types of melanocytes. None of the melanocytes showed any cellular toxicity or induction of apoptosis in response to UVR (morphological observation).

In our study, the number of melanocytes at 72 h post UVR (T_UVR_) was not lower than the number of melanocytes at the time of UV exposure (T_0_), though in comparison with growth of the untreated control group (T_Con_) reflects a decrease. This observation reiterates that UVR dose used in our study was not cytotoxic and hence no alteration was observed in the p53 expression. However, UVR-induced cytogenetic damage observed (MN, MLTN and PMN formation) in the White and Hispanic skin melanocytes did not trigger p53 activation. All these observations were also reported previously in a study which demonstrated that melanocytes can be resistant to UVR-induced apoptosis and melanocytes also have a lower ability (compared to unpigmented cells) to activate the p53 pathway in response to UVR [61].

There is very limited information available on the Sun exposure patterns and Sun protective practices in Hispanics. Such information will be useful to understand UVR as a risk factor for reported higher incidence rates of CM in Hispanics. Moreover, there exists a general perception of low risk of CM in Hispanics and Blacks due to their greater cutaneous pigmentation. This assumption perhaps has resulted in a delayed diagnosis, advanced stage at presentation, and a poor prognosis of CM amongst Hispanics and Blacks [58]. The results from our study unequivocally suggest that Hispanic skin (particularly lightly pigmented) is susceptible to UVR-induced cytogenetic damage that can trigger further molecular changes leading to CM. This study provides a benchmark for further investigation on the DNA damaging effects of chronic UVR exposure as risk for CM in Hispanics. Our results of UVR-induced alterations towards risk of CM presented here should facilitate implementation of improved preventive measures and public awareness directed towards the color of skin population.

## 4. Experimental Section

### 4.1. Melanocyte Culture 

Normal melanocyte primary cell lines, purchased from Coriell Cell Repositories (Camden, NJ, USA), used as the cell culture models were derived from 1-day old neonatal foreskin of subjects representing three ethnic groups: White-GM22250, Hispanic-GM22253, and Black-GM22258. All three cell lines were maintained in MGM-4 complete culture media (Lonza, Walkersville, MD, USA) in a humidified incubator at 37.5 °C, 5% CO_2_ atmosphere and fed at 48 h intervals. To ensure biological reproducibility, melanocyte cultures between passages 10 and 15 were used for all the experiments. The population doubling time (PDT) for White and Hispanic skin melanocytes is 32 h and 31 h respectively, while for Black skin melanocytes, PDT is 43 h. PDT was calculated from growth curves initiated at a seeding density of 10^5^ cells for each cell line. Experiments were initiated by plating cells either in 4-well glass chamber slides or 60 mm dishes. Cell growth was measured as alteration in cell number (hemocytometer cell count). Phase contrast images were acquired using an Eclipse TS100 microscope (Nikon Instruments Inc., Melville, NY, USA).

### 4.2. Melanocyte Exposure to UVR

The UVR source included two UVB broadband lamps (PLS-9W/12, Philips), obtained from Solarc Systems Inc. (Ontario, ON, Canada), with UV emission spectrum of UVB-68%, UVA2-22%, UVA1-9% & UVC < 1% (provided by the vendor and by using Simpson’s Rule). This UVR covers the reported environmental UV spectrum from the Sun [82]. Each melanocyte cell line was plated at a density of 10^5^ cells per well in a 4-well glass chamber slide, or 10^6^ cells per 60 mm dish; and cells were allowed to grow for 72 h to attain the log phase of growth. Melanocyte cultures were exposed to a single dose of 200 mJ/cm^2^ of UVR (media was replaced with PBS for treatment). The total spent media was collected from melanocyte cultures growing in 60 mm dishes; attached melanocytes were trypsinized, counted and pelleted to be stored at −20 °C for melanin estimation at T_0_ (time at UV exposure). The UV exposed melanocytes and the corresponding non-exposed controls were fed fresh media and were cultured further either for 24 h or 72 h time points for post UVR response. The total spent media was collected from UV exposed melanocyte cultures growing in 60 mm dishes at 24 h and 72 h post UVR and attached melanocytes were trypsinized, counted and pelleted to be stored at −20 °C for melanin estimation. Parallel experiments with melanocyte cultures in 4 well slides were fixed (4% paraformaldehyde) at 24 h and 72 h post UVR and stored at 4–8 °C for morphological analysis.

### 4.3. Hematoxylin (H)/Eosin (E) and Immunohistochemical (IHC) Staining 

One set of 4-well slides with fixed melanocytes were processed by standard histology staining protocol for HE to observe and quantify melanocytes containing MN, MLTN and PMN. Other set of 4-well slides were processed for IHC staining using our standardized protocol. Briefly, fixed melanocytes were washed in PBS, blocked in 1% BSA for 45 min, and antigen retrieved in 0.1% Triton-X for 20 min. Melanocytes were incubated overnight at 4–8 °C with appropriate dilutions of primary antibody Ki67 (#9449; Cell Signaling Technology, Danvers, MA, USA; 1:400) and Cyclin D1 (sc-8396; Santa Cruz Biotechnology, Dallas, TX, USA; 1:500) as proliferation markers and p53 (sc-263; Santa Cruz Biotechnology; 1:800) as apoptotic marker. Melanocytes were further incubated with biotinylated secondary antibody (Vector Laboratories, Burlingame, CA, USA) for 1 h, and Ki67, Cyclin D1, and p53 were visualized using the ABC reagent and AEC chromogen (Vector Laboratories) together with counter-stain with Hematoxylin. Expression of Ki67, Cyclin D1, and p53 was documented and images were acquired using a Nikon Eclipse 80i microscope.

### 4.4. Fontana-Masson Staining for Melanin 

The 4-well slides with fixed melanocytes were processed following vendor’s protocol (Fontana-Masson kit, Abcam, Cambridge, MA, USA). Melanocytes containing melanosomes with melanin was documented and images were acquired using a Nikon Eclipse 80i microscope.

### 4.5. Melanin Estimation 

Intracellular and released melanin from melanocyte cultures was extracted by solubilizing frozen cell pellets in 1 mL of 1 N NaOH and heated at 80 °C for 2 h. The samples were then centrifuged at 12,000× *g* for 10 min at RT and the supernatants were transferred to fresh tubes. The melanin content in these supernatants was measured spectrophotometrically at an absorbance of 470 nm against a standard curve of known concentrations of synthetic melanin (Sigma-Aldrich, St. Louis, MO, USA).

## 5. Conclusions

In conclusion, the results of the study presented here suggests that the risk of developing UVR-induced CM in any skin type is correlated with the level of cutaneous melanin pigment and the genetic background rather than just their ethnicity. Therefore, the risk of developing UVR-induced CM in Hispanics can be a consequence of the vast variation in their skin pigmentation that exists among this population. Overall, lightly pigmented Hispanic skin is at risk of developing UVR-induced CM. This study provides a benchmark for further investigation on the damaging effects of UVR as risk for CM in Hispanics.

## Supplementary Files

Supplementary File 1

## References

[1] Dellinger R.W., Liu-Smith F., Meyskens F.L. (2014). Continuing to illuminate the mechanisms underlying UV-mediated melanomagenesis. J. Photochem. Photobiol. B Biol..

[2] Jhappan C., Noonan F.P., Merlino G. (2003). Ultraviolet radiation and cutaneous malignant melanoma. Oncogene.

[3] Gray-Schopfer V., Wellbrock C., Marais R. (2007). Melanoma biology and new targeted therapy. Nature.

[4] Ries L., Melbert D., Krapcho M., Stinchcomb D., Howlander N., Horner M., Mariotto A., Miller B., Feuer E., Altekruse S. SEER Cancer Statistics Review, 1975–2014.

[5] Rouhani P., Hu S., Kirsner R.S. (2008). Melanoma in hispanic and black Americans. Cancer Control.

[6] Hu S., Parmet Y., Allen G., Parker D.F., Ma F., Rouhani P., Kirsner R.S. (2009). Disparity in melanoma: A trend analysis of melanoma incidence and stage at diagnosis among whites, Hispanics, and blacks in Florida. Arch. Dermatol..

[7] International Agency for Research on Cancer. http://ci5.iarc.fr/CI5plus/Pages/graph4_sel.aspx.

[8] D’Ischia M., Wakamatsu K., Napolitano A., Briganti S., Garcia-Borron J.C., Kovacs D., Meredith P., Pezzella A., Picardo M., Sarna T. (2013). Melanins and melanogenesis: Methods, standards, protocols. Pigment Cell Melanoma Res..

[9] Jimbow K., Quevedo W.C., Fitzpatrick T.B., Szabo G. (1976). Some aspects of melanin biology: 1950–1975. J. Investig. Dermatol..

[10] Solano F. (2014). Melanins: Skin Pigments and Much More—Types, Structural Models, Biological Functions, and Formation Routes. New J. Sci..

[11] Costin G.-E., Hearing V.J. (2007). Human skin pigmentation: Melanocytes modulate skin color in response to stress. FASEB J..

[12] Agar N., Young A.R. (2005). Melanogenesis: A photoprotective response to DNA damage?. Mutat. Res. Fundam. Mol. Mech. Mutagen..

[13] Brash D.E. (1997). Sunlight and the onset of skin cancer. Trends Genet..

[14] Garland C.F., Garland F.C., Gorham E.D. (2003). Epidemiologic evidence for different roles of ultraviolet A and B radiation in melanoma mortality rates. Ann. Epidemiol..

[15] Williams M., Ouhtit A. (2005). Towards a better understanding of the molecular mechanisms involved in sunlight-induced melanoma. J. Biomed. Biotechnol..

[16] Gandini S., Sera F., Cattaruzza M.S., Pasquini P., Abeni D., Boyle P., Melchi C.F. (2005). Meta-analysis of risk factors for cutaneous melanoma: I. Common and atypical naevi. Eur. J. Cancer.

[17] Tucker M.A. (2008). Is sunlight important to melanoma causation?. Cancer Epidemiol. Biomark. Prev..

[18] Pfeifer G.P., Besaratinia A. (2012). UV wavelength-dependent DNA damage and human non-melanoma and melanoma skin cancer. Photochem. Photobiol. Sci..

[19] Kaidbey K.H., Agin P.P., Sayre R.M., Kligman A.M. (1979). Photoprotection by melanin—A comparison of black and Caucasian skin. J. Am. Acad. Dermatol..

[20] Ortonne J.-P. (2002). Photoprotective properties of skin melanin. Br. J. Dermatol..

[21] Wei Q., Lee J.E., Gershenwald J.E., Ross M.I., Mansfield P.F., Strom S.S., Wang L.-E., Guo Z., Qiao Y., Amos C.I. (2003). Repair of UV light-induced DNA damage and risk of cutaneous malignant melanoma. J. Natl. Cancer Inst..

[22] Hauser J.E., Kadekaro A.L., Kavanagh R.J., Wakamatsu K., Terzieva S., Schwemberger S., Babcock G., Rao M.B., Ito S., Abdel-Malek Z.A. (2006). Melanin content and MC1R function independently affect UVR-induced DNA damage in cultured human melanocytes. Pigment Cell Res..

[23] Meyle K.D., Guldberg P. (2009). Genetic risk factors for melanoma. Hum. Genet..

[24] Cao J., Wan L., Hacker E., Dai X., Lenna S., Jimenez-Cervantes C., Wang Y., Leslie N., Xu G., Widlund H. (2013). MC1R is a potent regulator of PTEN after UV exposure in melanocytes. Mol. Cell.

[25] Davies H., Bignell G.R., Cox C., Stephens P., Edkins S., Clegg S., Teague J., Woffendin H., Garnett M.J., Bottomley W. (2002). Mutations of the BRAF gene in human cancer. Nature.

[26] Platz A., Egyhazi S., Ringborg U., Hansson J. (2008). Human cutaneous melanoma; a review of NRAS and BRAF mutation frequencies in relation to histogenetic subclass and body site. Mol. Oncol..

[27] Curtin J.A., Busam K., Pinkel D., Bastian B.C. (2006). Somatic activation of KIT in distinct subtypes of melanoma. J. Clin. Oncol..

[28] Puri N., Ahmed S., Janamanchi V., Tretiakova M., Zumba O., Krausz T., Jagadeeswaran R., Salgia R. (2007). c-Met is a potentially new therapeutic target for treatment of human melanoma. Clin. Cancer Res..

[29] Chattopadhyay C., Ellerhorst J.A., Ekmekcioglu S., Greene V.R., Davies M.A., Grimm E.A. (2012). Association of activated c-Met with NRAS-mutated human melanomas: A possible avenue for targeting. Int. J. Cancer.

[30] Kuluncsics Z., Perdiz D., Brulay E., Muel B., Sage E. (1999). Wavelength dependence of ultraviolet-induced DNA damage distribution: Involvement of direct or indirect mechanisms and possible artefacts. J. Photochem. Photobiol. B Biol..

[31] Korytowski W., Pilas B., Sarna T., Kalyanaraman B. (1987). Photoinduced generation of hydrogen peroxide and hydroxyl radicals in melanins. Photochem. Photobiol..

[32] Cadet J., Berger M., Douki T., Morin B., Raoul S., Ravanat J., Spinelli S. (1997). Effects of UV and visible radiation on DNA-final base damage. Biol. Chem..

[33] Bennett D.C. (2008). Ultraviolet wavebands and melanoma initiation. Pigment Cell Melanoma Res..

[34] Mouret S., Baudouin C., Charveron M., Favier A., Cadet J., Douki T. (2006). Cyclobutane pyrimidine dimers are predominant DNA lesions in whole human skin exposed to UVA radiation. Proc. Natl. Acad. Sci. USA.

[35] Premi S., Wallisch S., Mano C.M., Weiner A.B., Bacchiocchi A., Wakamatsu K., Bechara E.J.H., Halaban R., Douki T., Brash D.E. (2015). Chemiexcitation of melanin derivatives induces DNA photoproducts long after UV exposure. Science.

[36] Bradley M. (1981). Double-strand breaks in DNA caused by repair of damage due to ultraviolet light. J. Supramol. Struct. Cell Biochem..

[37] Emri G., Wenczl E., van Erp P., Jans J., Roza L., Horkay I., Schothorst A.A. (2000). Low doses of UVB or UVA induce chromosomal aberrations in cultured human skin cells. J. Investig. Dermatol..

[38] De Gruijl F.R., van Kranen H.J., Mullenders L.H.F. (2001). UV-induced DNA damage, repair, mutations and oncogenic pathways in skin cancer. J. Photochem. Photobiol. B Biol..

[39] Tadokoro T., Kobayashi N., Zmudzka B.Z., Ito S., Wakamatsu K., Yamaguchi Y., Korossy K.S. (2003). UV-induced DNA damage and melanin content in human skin differing in racial/ethnic origin. FASEB J..

[40] Bustamante J., Bredeston L., Malanga G., Mordoh J. (1993). Role of melanin as a scavenger of active oxygen species. Pigment Cell Res..

[41] Kvam E., Tyrrell R.M. (1999). The role of melanin in the induction of oxidative DNA base damage by ultraviolet A irradiation of DNA or melanoma cells. J. Investig. Dermatol..

[42] Kvam E., Dahle J. (2004). Melanin synthesis may sensitize melanocytes to oxidative DNA damage by ultraviolet A radiation and protect melanocytes from direct DNA damage by ultraviolet B radiation. Pigment Cell Res..

[43] Wang H.-T., Choi B., Tang M. (2010). Melanocytes are deficient in repair of oxidative DNA damage and UV-induced photoproducts. Proc. Natl. Acad. Sci. USA.

[44] Budden T., Bowden N.A. (2013). The role of altered nucleotide excision repair and UVB-induced DNA damage in melanomagenesis. Int. J. Mol. Sci..

[45] Nickerson J.A. (1998). Nuclear dreams: The malignant alteration of nuclear architecture. J. Cell. Biochem..

[46] Zink D., Fischer A.H., Nickerson J.A. (2004). Nuclear structure in cancer cells. Nat. Rev. Cancer.

[47] Heddle J.A., Hite M., Kirkhart B., Mavournin K., MacGregor J.T., Newell G.W., Salamone M.F. (1983). The induction of micronuclei as a measure of genotoxicity. A report of the U.S. Environmental Protection Agency Gene-Tox Program. Mutat. Res..

[48] Roser M., Bohm A., Oldigs M., Weichenthal M., Reimers U., Schmidt-Preuss U., Breitbart E.W., Rudiger H.W. (1989). Ultraviolet-induced formation of micronuclei and sister chromatid exchange in cultured fibroblasts of patients with cutaneous malignant melanoma. Cancer Genet. Cytogenet..

[49] Fenech M., Kirsch-Volders M., Natarajan A.T., Surralles J., Crott J.W., Parry J., Norppa H., Eastmond D.A., Tucker J.D., Thomas P. (2011). Molecular mechanisms of micronucleus, nucleoplasmic bridge and nuclear bud formation in mammalian and human cells. Mutagenesis.

[50] Zusman I., Kozlenko M., Zimber A. (1991). Nuclear polymorphism and nuclear size in precarcinomatous and carcinomatous lesions in rat colon and liver. Cytometry.

[51] Cornforth M.N., Goodwin E.H. (1991). Transmission of radiation-induced acentric chromosomal fragments to micronuclei in normal human fibroblasts. Radiat. Res..

[52] Hu S., Ma F., Collado-Mesa F., Kirsner R.S. (2004). UV radiation, latitude, and melanoma in U.S. Hispanics and blacks. Arch. Dermatol..

[53] Cockburn M.G., Zadnick J., Deapen D. (2006). Developing epidemic of melanoma in the Hispanic population of California. Cancer.

[54] Sullivan M.G. (2006). Thick melanoma lesions increasing in hispanics. Clin. Rounds.

[55] Pollitt R.A., Clarke C.A., Swetter S.M., Peng D.H., Zadnick J., Cockburn M. (2011). The expanding melanoma burden in California hispanics: Importance of socioeconomic distribution, histologic subtype, and anatomic location. Cancer.

[56] Clairwood M., Ricketts J., Grant-kels J., Gonsalves L. (2014). Melanoma in skin of color in Connecticut: An analysis of melanoma incidence and stage at diagnosis in non-Hispanic blacks , non-Hispanic whites, and Hispanics. Int. J. Dermatol..

[57] Jaimes N., Oliveria S., Halpern A. (2013). A cautionary note on melanoma screening in the Hispanic/Latino population. JAMA Dermatol..

[58] Wu X.C., Eide M.J., King J., Saraiya M., Huang Y., Wiggins C., Barnholtz-Sloan J.S., Martin N., Cokkinides V., Miller J. (2011). Racial and ethnic variations in incidence and survival of cutaneous melanoma in the United States, 1999–2006. J. Am. Acad. Dermatol..

[59] Scholzen T., Gerdes J. (2000). The Ki-67 protein: From the known and the unknown. J. Cell. Physiol..

[60] Knudsen K.E., Diehl J.A., Haiman C., Knudsen E.S. (2006). Cyclin D1: Polymorphism, aberrant splicing and cancer risk. Oncogene.

[61] Marrot L., Belaïdi J.-P., Jones C., Perez P., Meunier J.-R. (2005). Molecular responses to stress induced in normal human caucasian melanocytes in culture by exposure to simulated solar UV. Photochem. Photobiol..

[62] Barker D., Dixon K., Medrano E.E., Smalara D., Im S., Mitchell D., Babcock G., Abdel-malek Z.A. (1995). Comparison of the responses of human melanocytes with different melanin contents to ultraviolet B irradiation. Cancer Res..

[63] Medrano E.E., Im S., Yang F. (1995). Ultraviolet B light induces G1 arrest in human melanocytes by prolonged inhibition of retinoblastoma protein phosphorylation associated with long-term expression of the p21 Waf-1/SDI-1/Cip-1 protein ultraviolet B light Induces G, arrest in human mel. Cancer.

[64] Wenczl E., van der Schans G.P., Roza L., Kolb R.M., Timmerman A.J., Smit N.P.M., Pavel S., Schothorst A.A. (1998). (Pheo)melanin photosensitizes VA-induced DNA damage in cultured human melanocytes. J. Investig. Dermatol..

[65] Marrot L., Belaidi J.P., Meunier J.R., Perez P., Agapakis-Causse C. (1999). The human melanocyte as a particular target for UVA radiation and an endpoint for photoprotection assessment. Photochem. Photobiol..

[66] Hill H.Z., Hill G.J. (2000). UVA, pheomelanin and the carcinogenesis of melanoma. Pigment Cell Res..

[67] Besaratinia A., Yoon J.-I., Schroeder C., Bradforth S.E., Cockburn M., Pfeifer G.P. (2011). Wavelength dependence of ultraviolet radiation-induced DNA damage as determined by laser irradiation suggests that cyclobutane pyrimidine dimers are the principal DNA lesions produced by terrestrial sunlight. FASEB J..

[68] Fitzpatrick T.B., Szabo G. (1959). The melanocyte: CYTOLOGY and cytochemistry. J. Investig. Dermatol..

[69] Toda K., Pathak M., Parrish J.A., Fitzpatrick T.B., Quevedo W.C. (1972). Alteration of racial differences in melanosome distribution in human epidermis after exposure to ultraviolet light. Nat. New Biol..

[70] Sturm R.A., Box N.F., Ramsay M. (1998). Human pigmentation genetics: The difference is only skin deep. BioEssays.

[71] Brenner M., Hearing V.J. (2008). The protective role of melanin against UV damage in human skin. Photochem. Photobiol..

[72] Eller M.S., Ostrom K., Gilchrest B.A. (1996). DNA damage enhances melanogenesis. Proc. Natl. Acad. Sci. USA.

[73] Eller M.S., Gilchrest B.A. (2000). Tanning as part of the eukaryotic SOS response. Pigment Cell Res..

[74] Duval C., Régnier M., Schmidt R. (2001). Distinct melanogenic response of human melanocytes in mono-culture, in co-culture with keratinocytes and in reconstructed epidermis, to UV exposure. Pigment Cell Res..

[75] Virador V.M., Muller J., Wu X., Abdel-Malek Z.A., Yu Z.X., Ferrans V.J., Kobayashi N., Wakamatsu K., Ito S., Hammer J.A. (2002). Influence of alpha-melanocyte-stimulating hormone and ultraviolet radiation on the transfer of melanosomes to keratinocytes. FASEB J..

[76] Hall P., McKee P., Menage H.D., Dover R., Lane D.P. (1993). High levels of p53 protein in UV-irradiated normal human skin. Oncogene.

[77] Yamaguchi Y., Coelho S.G., Zmudzka B.Z., Takahashi K., Beer J.Z., Hearing V.J., Miller S.A. (2008). Cyclobutane pyrimidine dimer formation and p53 production in human skin after repeated UV irradiation. Exp. Dermatol..

[78] Ullrichs S.J., Anderson C.W., Mercern W.E. (1992). The p53 Tumor Suppressor Protein, a Modulator of Cell Proliferation. J. Biol. Chem..

[79] Bálint E.E., Vousden K.H. (2001). Activation and activities of the p53 tumour suppressor protein. Br. J. Cancer.

[80] Kastan M.B., Onyekwere O., Sidransky D., Vogelstein B., Craig R.W. (1991). Participation of p53 protein in the cellular response to DNA damage. Cancer Res..

[81] Eller M.S., Maeda T., Magnoni C., Atwal D., Gilchrest B.A. (1997). Enhancement of DNA repair in human skin cells by thymidine dinucleotides: Evidence for a p53-mediated mammalian SOS response. Proc. Natl. Acad. Sci. USA.

[82] Diffey B.L. (2002). Sources and measurement of ultraviolet radiation. Methods.

