# Supplementary material for: Ultraviolet Radiation-Induced Cytogenetic Damage in White, Hispanic and Black Skin Melanocytes: A Risk for Cutaneous Melanoma
Source: Cancers (Basel). 2015 Aug 14;7(3):1586–604. doi: 10.3390/cancers7030852 (PMC4586785; doi:10.3390/cancers7030852)
Supplement: Supplementary File 1 [file cancers-07-00852-s001.pdf]

Supplementary Material

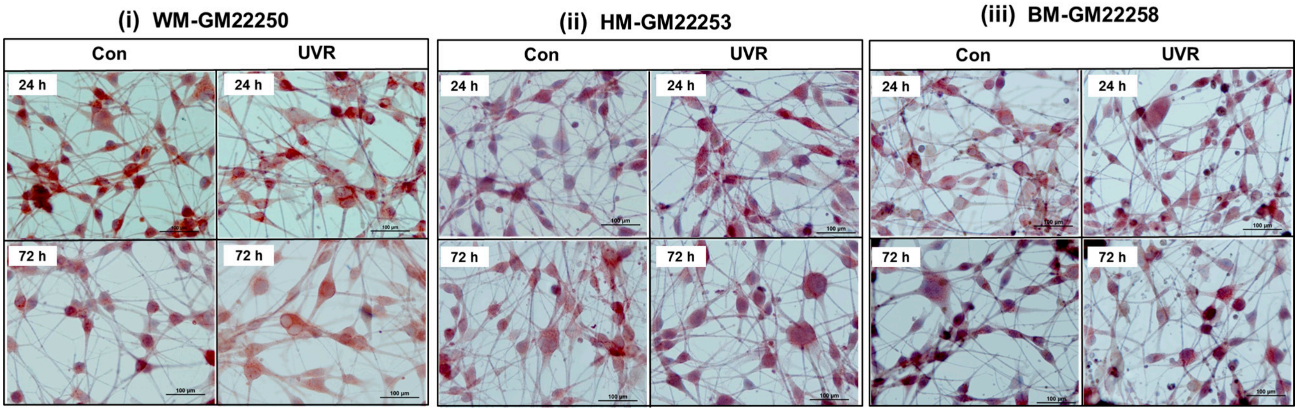

Figure S1. Effect of UVR on the expression of apoptosis marker, p53 on normal skin melanocytes; (**i**) White, (**ii**) Hispanic and (**iii**) Black at 24 h and 72 h post UV exposure. Images were obtained at 40×.

© 2015 by the authors; licensee MDPI, Basel, Switzerland. This article is an open access article distributed under the terms and conditions of the Creative Commons Attribution license (http://creativecommons.org/licenses/by/4.0/).
